# A Nomogram to Predict Adequate Lymph Node Recovery before Resection of Colorectal Cancer

**DOI:** 10.1371/journal.pone.0168156

**Published:** 2016-12-16

**Authors:** Zhen-yu Zhang, Cong Li, Wei Gao, Xiao-wei Yin, Qi-feng Luo, Nan Liu, Shiva Basnet, Zhen-ling Dai, Hai-yan Ge

**Affiliations:** 1 Department of Gastrointestinal Surgery, Shanghai East Hospital, Tongji University School of Medicine, Shanghai, China; 2 Department of Spine Surgery, Shanghai East Hospital, Tongji University School of Medicine, Shanghai, China; 3 Department of General Surgery, Qingpu Branch of Zhongshan Hospital, Fudan University, Shanghai, China; Institut national de la recherche scientifique, CANADA

## Abstract

Increased lymph node count (LNC) has been associated with prolonged survival in colorectal cancer (CRC), but the underlying mechanisms are still poorly understood. The study aims to identify new predictors and develop a preoperative nomogram for predicting the probability of adequate LNC (≥ 12). 501 eligible patients were retrospectively selected to identify clinical-pathological factors associated with LNC ≥ 12 through univariate and multivariate logistic regression analyses. The nomogram was built according to multivariate analyses of preoperative factors. Model performance was assessed with concordance index (c-index) and area under the receiver operating characteristic curve (AUC), followed by internal validation and calibration using 1000-resample bootstrapping. Clinical validity of the nomogram and LNC impact on stage migration were also evaluated. Multivariate analyses showed patient age, CA19-9, circulating lymphocytes, neutrophils, platelets, tumor diameter, histology and deposit significantly correlated with LNC (*P* < 0.05). The effects were marginal for CEA, anemia and CRC location (0.05 < *P* < 0.1). The multivariate analyses of preoperative factors suggested decreased age, CEA, CA19-9, neutrophils, proximal location, and increased platelets and diameter were significantly associated with increased probability of LNC ≥ 12 (*P* < 0.05). The nomogram achieved c-indexes of 0.75 and 0.73 before and after correction for overfitting. The AUC was 0.75 (95% CI, 0.70–0.79) and the clinically valid threshold probabilities were between 10% and 60% for the nomogram to predict LNC < 12. Additionally, increased probability of adequate LNC before surgery was associated with increased LNC and negative lymph nodes rather than increased positive lymph nodes, lymph node ratio, pN stages or AJCC stages. Collectively, the results indicate the LNC is multifactorial and irrelevant to stage migration. The significant correlations with preoperative circulating markers may provide new explanations for LNC-related survival advantage which is reflected by the implication of regional and systemic antitumor immune responses.

## Introduction

Lymph node count (LNC) is an important parameter of routine pathological report after resection of colorectal cancer (CRC) [1, 2]. Adequacy of lymph node assessment is usually required for accurate staging of patients with CRC [3, 4]. This goal has been simplified in clinical practice as a dedicated service of recovering more lymph nodes to meet a minimum requirement of 12 lymph nodes, as recommended by the American Joint Committee on Cancer (AJCC) and College of American Pathologists [5, 6]. Although many studies have demonstrated a correlation between increased LNC and prolonged survival in patients with CRC [7–11], the underlying mechanisms of LNC-associated survival advantage are still poorly understood [3, 12].

Several mechanisms have been proposed to explain LNC-related impact on survival. The most common mechanism is stage migration [7, 12, 13]. Theoretically, the likelihood of being under-staged and erroneously treated is reduced for patients with more lymph nodes recovered [7]. Accurate staging is essential for selection of appropriate therapy and maximization of survival benefit. However, recent studies suggest that increased LNC does not accompany with significant migration of pN stages [14, 15]. Another study also shows that pathological reevaluations for patients with less than 12 lymph nodes identified after initial assessment does not lead to marked increase in metastatic lymph nodes, lymph node ratio (LNR) or obvious migration of pN stages [16]. The second considered mechanism is extended lymphadenectomy [3, 12]. Complete mesocolic excision (CME) removes more lymph nodes, increasing the chance of eliminating skip metastasis and micrometastsis in lymph nodes at a longer distance from the primary tumor [12, 17]. A survival benefit for colon cancer patients after CME has been observed in several studies [17, 18], whilst conclusive evidence by clinical trials remains absent [19]. The third mechanism which exhibits appropriate rationality is that some predictors for increased lymph nodes are also independent factors for a survival advantage in CRC [3, 12]. For instance, younger age and microsatellite instable (MSI) phenotype are associated with more lymph nodes as well as better outcomes in CRC patients [20–22]. However, it is also reported that larger number of lymph node is more frequently observed in CRC with a greater diameter, poor histology, a proximal location and a deeper depth of penetration as well [3, 12, 13]. These factors tend to exert adverse rather than favorable effects on survival of patients with CRC [3, 23]. The contradiction indicates that the relationship between increased LNC and prolonged survival may be not straightforward as expected. Investigations on new responsible markers may help to understand the survival benefit.

Preoperative serum tumor markers and blood cell counts are routinely used to evaluate surgically-treated patients with CRC [3]. The relationship between them and LNC remains unclear. In this study, we retrospectively reviewed these preoperative parameters in addition to demographical and pathological characteristics of CRC patients who received curative surgery at the department of gastrointestinal surgery, Shanghai East Hospital. The first aim of the study is to identify potential clinical-pathological correlators for the benchmark of 12 lymph nodes in the Chinese population. The second aim is to develop and internal validate a nomogram to predict the possibility of inadequate lymph node recovery (LNC < 12) before curative surgery. The study is expected to provide new traces for causative mechanisms that may explain associations between increased LNC and survival, and help to enable an adequate preoperative assessment, preparation and appropriate clinical decision for individualized therapy with the LNC-predictive nomogram.

## Materials and Methods

### Patients and variables

Medical records of 690 consecutive patients with primary CRC, who received surgery with a curative intent at the department of gastrointestinal surgery of Shanghai East Hospital between August 2011 and June 2015, were retrospectively reviewed. Patients were considered for subsequent analysis based on the following exclusion criteria: (1) patient who underwent emergency surgeries (n = 6), (2) patients who received chemo-radiotherapy before blood tests and surgeries (n = 19), (3) pTis disease or multiple primaries (n = 13), (4) pathologically proven non-R0 resection (n = 8), and (5) patients with incomplete or inconsistent data to specify any used variables (n = 143).

Both preoperative and pathological variables were accessed for each patient. Preoperative variables included sex, age, white blood cell count (WBC, reference: 3.7–9.2 × 10^6^/mL), neutrophil count (NEU, reference: 2.0–7.0 × 10^6^/mL), lymphocyte count (LYM, reference: 0.8–4.0 × 10^6^/mL), platelet count (PLT, 10^6^/mL), hemoglobin concentration (Hb, g/L), serum carcinoembryonic antigen (CEA, reference: < 5.2 ng/mL), CA19-9 (reference, < 37 U/mL), CA50 (reference, < 25 U/mL), CA72-4 (reference, < 6.9 U/mL), type of surgery (open surgery or laparoscopy), tumor location and maximum diameter indicated by preoperative computed tomography scan. Pathological parameters assessed were experience of pathologist (junior or senior title), tumor macroscopy, grade, histology, vascular invasion, perineural invasion, LNC and tumor deposit in addition to tumor-node-metastasis classification by the 7th edition AJCC cancer stating manual. Presence of anemia was defined as a Hb concentration ≤ 110 g/L for females or 120 g/L for males. The normal upper limit of PLT used was 320 × 10^6^/mL for both sexes to define thrombocytosis. Laparoscopy was only implemented for patients who were appropriate and willing to receive it.

### Statistical methods

Continuous variables were compared with parametric or nonparametric methods depending on the distribution of the data. Discontinuous variables were compared using Chi-squared test or Fisher exact test. Concentrations of blood markers were pre-categorized to predict LNC ≥ 12 according to their normal references (i.e., for Hb and PLT) or optimal cut-off values determined by maximization of Yuden Index with receiver operating characteristic (ROC) curve analysis, based on clinical reasoning and significance. To identify correlative factors for LNC ≥ 12, significant variables in univariate logistic regression analysis were evaluated in multivariate logistic analysis with a stepwise forward elimination of insignificant variables using the PASW 18.0 program (SPSS, Chicago, IL). To identify predictors for LNC ≥ 12 that would be used in the nomogram, similar univariate and multivariate logistic analyses were performed using preoperative variables to derive a final variable formula. Nomogram was built in R program (v 3.2.3) with the *rms* package. Performance of the nomogram was assessed by concordance index (c-index) and the area under the ROC curve (AUC) with associated 95% confident interval (95% CI). Internal validation and calibration of the nomogram were conducted by 1000-resample bootstrapping. The ranges of threshold probability, between which the nomogram was clinically valid, were determined by decision curve analysis (DCA). After addressing model performance, each patient was given an aggregated point on the basis of the nomogram. The points of all patients were then divided into quartiles to investigate whether increased probability of adequate lymph node recovery had significant impact on diagnosis of nodal status as well as AJCC stages. Statistical significance of all tests was set as a two-sided *P* value < 0.05.

The study data were extracted and analyzed in January of 2016 under the condition of anonymity, during which the researchers did not have the access to patient-identifying information. The study adhered to the Declaration of Helsinki for medical research involving human subjects [24] and the STROBE guidelines (Please refer to S1 Table for STROBE guidelines). Due to difficulties to be obtained retrospectively, informed consents from participants were waived upon the approval of the Ethics Committee of the Shanghai East Hospital (Study ID: 2015-LSD-070).

## Results

### Characteristics of the patients

A final set of 501 patients with CRC was selected. Related clinical-pathological characteristics were shown in Table 1.

**Table 1:** Characteristics of patients with colorectal cancer.

	Lymph node count		
Variables	<12/≥12 (n)	<12/≥12 (%)	*P*
Preoperative variables			
Age, year			0.007
Mean	69.6/66.8	—	
SD	10.4/11.3	—	
Sex			0.158
Female	58/139	29.4/70.6	
Male	108/196	35.5/64.5	
CEA, ng/mL			0.034
≤ 4.4	73/181	28.7/71.3	
> 4.4	93/154	37.7/62.3	
CA19-9, U/mL			0.028
≤ 6.7	29/88	24.8/75.2	
> 6.7	137/247	35.7/64.3	
CA50, U/mL			0.077
≤ 8.9	122/220	35.7/64.3	
> 8.9	44/115	27.7/72.3	
CA72-4, U/mL			0.086
≤ 1.2	45/68	39.8/60.2	
> 1.2	121/267	31.2/68.8	
Lymphocyte count, 10^6^/mL			0.035
≤ 1.2	30/89	25.2/74.8	
> 1.2	136/246	35.6/64.4	
White blood cell count, 10^6^/mL			0.191
≤ 6.7	112/206	35.2/64.8	
> 6.7	54/129	29.5/70.5	
Neutrophils, 10^6^/mL			0.018
≤ 2.2	6/32	15.8/84.2	
> 2.2	160/303	34.6/65.4	
Anemia			< 0.001
No	51/163	23.8/76.2	
Yes	115/172	40.1/59.9	
Platelet, 10^6^/mL			< 0.001
≤ 320	157/267	37.0/63.0	
> 320	9/68	11.7/88.3	
Location			0.003
Proximal colon	35/117	23.0/77.0	
Distal colon	53/74	41.7/58.3	
Rectum	78/144	35.1/64.9	
Diameter, cm			< 0.001
Mean	3.8/4.7	—	
SD	1.8/1.8	—	
Surgery			0.165
Open	141/299	32.0/68.0	
Laparoscopy	25/36	41.0/59.0	
Pathological variables			
Pathologist			0.799
Junior	24/59	28.9/71.1	
Senior	142/276	34.0/66.0	
Macroscopy			0.785
Polypoid type	70/137	33.8/66.2	
Ulcerative + Infiltrating type	96/198	32.7/67.3	
Differentiation			0.034
G1+G2	137/248	35.6/64.4	
G3+G4	29/87	25.0/75.0	
Histology			< 0.001
Adenocarcinoma	159/285	35.8/64.2	
Poorly differentiated + Mucous + Signet ring	7/50	12.3/87.7	
Lymphovascular invasion			0.775
No	85/167	33.7/66.3	
Yes	81/168	32.5/67.5	
Perineural invasion			0.239
No	95/210	31.1/68.9	
Yes	71/125	36.2/63.8	
Deposit			< 0.001
No	122/305	28.6/71.4	
Yes	44/30	59.5/40.5	
pT stage			0.009
T1	19/13	59.4/40.6	
T2	29/58	33.3/66.7	
T3	65/159	29.0/71.0	
T4	53/105	33.5/66.5	
PLNC			0.157
Median	0/0	—	
IQR	0-1/0-2	—	
pN stage			0.081
N0	90/180	33.3/66.7	
N1	63/107	37.1/62.9	
N2	13/48	21.3/78.7	
Metastasis			0.572
M0	155/317	32.8/67.2	
M1	11/18	37.9/62.1	

Abbreviations: SD, standard deviation; CEA, carcinoembryonic antigen; PLNC, positive lymph node count; IQR, interquartile range.

### Predictors for LNC ≥ 12

Results of univariate logistic regression analysis were shown in Table 2. Variables assessed in multivariate analysis and significant variables with associated odds ratio (OR) were shown in Table 3. In the multivariate analysis (Model A in Table 3) which adjusted both preoperative and pathological factors, patient age, CA19-9, LYM, NEU, PLT, tumor diameter, histology and tumor deposit emerged to be relatively important factors (*P* < 0.05). A marginal effect was seen with respect to CEA, anemia and location of CRC (*P* > 0.05 and *P* < 0.1). In the multivariate analysis (Model B in Table 3) which adjusted only preoperative factors, decreased age, CEA, CA19-9, NEU, proximal location, increased PLT and tumor diameter were associated with increased probability of LNC ≥ 12 with a more prominent predictive effect (*P* < 0.05). These predictors were selected to establish the nomogram. Additionally, the effect of the presence of anemia remained marginal (*P* = 0.097).

**Table 2:** Univariate logistic regression analysis.

	Univariate analysis	
Predictors for LNC ≥ 12	OR (95% CI)	*P* value
Preoperative variables		
Age, year	0.98 (0.96–0.99)	0.008
Sex (Male v Female)	0.76 (0.51–1.11)	0.158
CEA (> 4.4 v ≤ 4.4)	0.67 (0.46–0.97)	0.035
CA19-9 (> 6.7 v ≤ 6.7)	0.59 (0.37–0.95)	0.029
CA50 (> 8.9 v ≤ 8.9)	1.45 (0.96–2.19)	0.077
CA72-4 (> 1.2 v ≤ 1.2)	1.46 (0.95–2.25)	0.087
Lymphocyte count (> 1.2 v ≤ 1.2)	0.61 (0.38–0.97)	0.037
White blood cell count (> 6.7 v ≤ 6.7)	1.30 (0.88–1.92)	0.191
Neutrophils (> 2.2 v ≤ 2.2)	0.36 (0.15–0.87)	0.023
Anemia (Yes v No)	0.47 (0.32–0.69)	< 0.001
Platelet (> 320 v ≤ 320)	4.44 (2.16–9.15)	< 0.001
Location (referent = Proximal colon)		
Distal colon	0.42 (0.25–0.70)	0.001
Rectum	0.55 (0.35–0.88)	0.013
Diameter, cm	1.37 (1.21–1.55)	< 0.001
Surgery (Laparoscopy v Open)	0.68 (0.39–1.17)	0.166
Pathological variables		
Pathologist (Senior v Junior)	0.79 (0.47–1.32)	0.372
Macroscopy (referent = Polypoid type)		
Ulcerative + Infiltrating type	1.05 (0.72–1.54)	0.785
Differentiation (referent = G1+G2)		
G3+G4	1.66 (1.04–2.65)	0.035
Histology (referent = Adenocarcinoma)		
Poorly differentiated + Mucous + Signet ring	3.98 (1.76–9.00)	0.001
Lymphovascular invasion (Yes v No)	1.06 (0.73–1.53)	0.775
Perineural invasion (Yes v No)	0.80 (0.55–1.16)	0.239
Deposit (Yes v No)	0.27 (0.16–0.45)	< 0.001
pT stage (referent = T1)		
T2	2.92 (1.27–6.73)	0.012
T3	3.58 (1.67–7.66)	0.001
T4	2.90 (1.33–6.31)	0.007
pN stage (referent = N0)		
N1	0.85 (0.57–1.27)	0.424
N2	1.85 (0.95–3.58)	0.070
Metastasis (M1 v M0)	0.80 (0.37–1.74)	0.572

Abbreviations: LNC, lymph node count; CEA, carcinoembryonic antigen; OR, odds ratio; 95% CI, 95% confident interval.

**Table 3:** Multivariate logistic regression analysis.

	Multivariate Model A		Multivariate Model B	
Predictors for LNC ≥ 12	OR (95% CI)	*P* value	OR (95% CI)	*P* value
Preoperative variables				
Age, year	0.98 (0.96–0.99)	0.019	0.98 (0.96–0.99)	0.012
CEA (> 4.4 v ≤ 4.4)	—	0.062	0.64 (0.42–0.97)	0.036
CA19-9 (> 6.7 v ≤ 6.7)	0.53 (0.32–0.89)	0.017	0.56 (0.34–0.93)	0.025
Lymphocyte count (> 1.2 v ≤ 1.2)	0.52 (0.31–0.88)	0.015	—	0.071
Neutrophils (> 2.2 v ≤ 2.2)	0.29 (0.11–0.76)	0.012	0.25 (0.10–0.62)	0.003
Anemia (Yes v No)	—	0.067	—	0.097
Platelet (> 320 v ≤ 320)	3.58 (1.63–7.88)	0.002	3.26 (1.50–7.08)	0.003
Location (referent = Proximal colon)		0.056		0.034
Distal colon	—	0.020	0.47 (0.27–0.83)	0.009
Rectum	—	0.385	0.67 (0.40–1.11)	0.122
Diameter, cm	1.37 (1.19–1.58)	< 0.001	1.38 (1.20–1.59)	< 0.001
Pathological variables				
Differentiation (referent = G1+G2)				
G3+G4	—	0.402	—	—
Histology (referent = Adenocarcinoma)				
Poorly differentiated + Mucous + Signet ring	3.42 (1.40–8.31)	0.007	—	—
Deposit (Yes v No)	0.23 (0.13–0.42)	< 0.001	—	—
pT stage (referent = T1)		0.412		
T2	—	0.740	—	—
T3	—	0.857	—	—
T4	—	0.347	—	—

Abbreviations: LNC, lymph node count; CEA, carcinoembryonic antigen; OR, odds ratio; 95% CI, 95% confident interval.

### Development of nomogram

**Fig 1:**
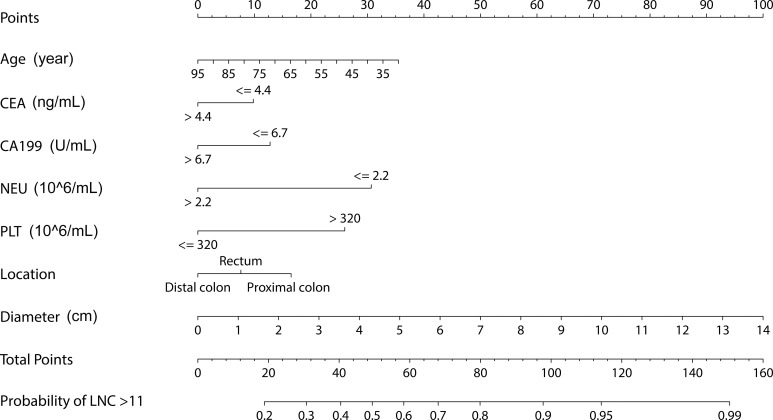
Nomogram to predict LNC > 11 before surgery. CEA, carcinoembryonic antigen; NEU, neutrophils; PLT, platelets.

The nomogram to predict the probability of LNC ≥ 12 for CRC patients before surgery was shown in Fig 1.

### Performance of nomogram

**Fig 2:**
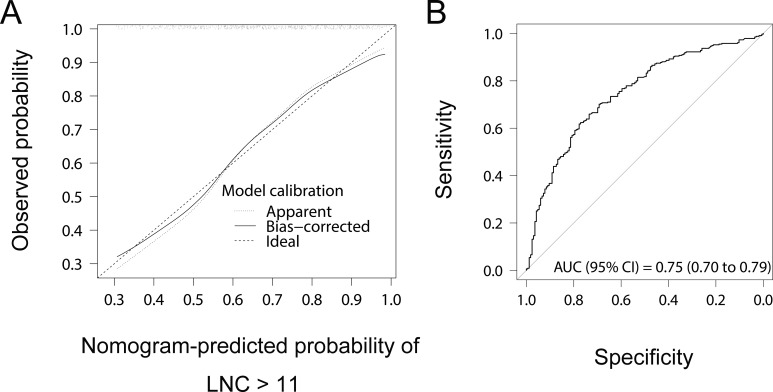
**(A) Calibration plot and (B) receiver operating characteristic (ROC) curve analysis.** LNC, lymph node count; AUC, the area under the ROC curve; 95% CI, 95% confident interval.

**Fig 3:**
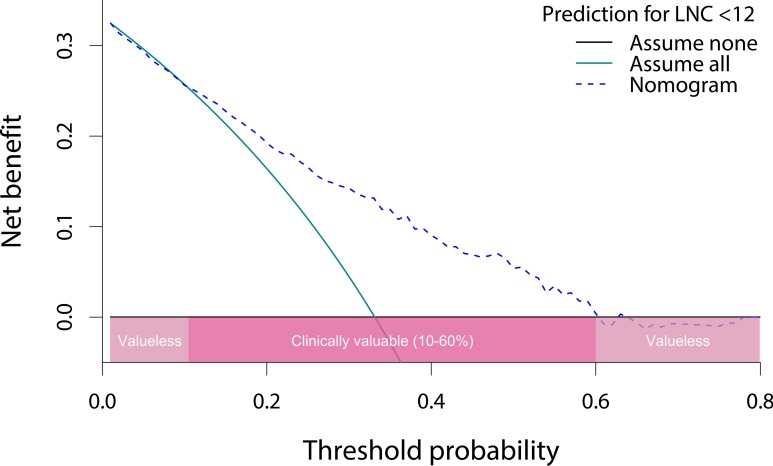
Decision curve analysis of the nomogram. LNC, lymph node count.

The c-index of the nomogram was 0.75 and 0.73 before and after correction for overfitting. The bootstrap calibration plot (Fig 2A) indicated a good agreement between nomogram-predicted and observed probability of adequate lymph node recovery. Moreover, the AUC of nomogram to predict LNC ≥ 12 was 0.75 (95% CI, 0.70–0.79), as estimated by the ROC curve analysis using nomogram-derived total points of patients as a predictive variable (Fig 2B). The results of DCA (Fig 3) suggested that the nomogram was clinically valid within probability thresholds between 10% and 60% when predicting the probability of failure to adequate lymph node recovery.

### Impact of increased probability of adequate lymph node recovery

Four subgroups were classified by dividing the nomogram-derived total scores of all patients into quartiles. The probability of adequate lymph node recovery significantly corrected with score quartiles. Compared with the 1st quartile, the highest odds ratio (OR) to predict the probability was associated with the 4th quartile (OR = 14.48, 95% CI = 7.35 to 28.54, *P* < 0.001), and the odds ratios were less dominant for the 2nd (OR = 2.79, 95% CI = 1.67 to 4.65, *P* < 0.001) and the 3rd (OR = 6.10, 95% CI = 3.49 to 10.66, *P* < 0.001) quartiles.

**Fig 4:**
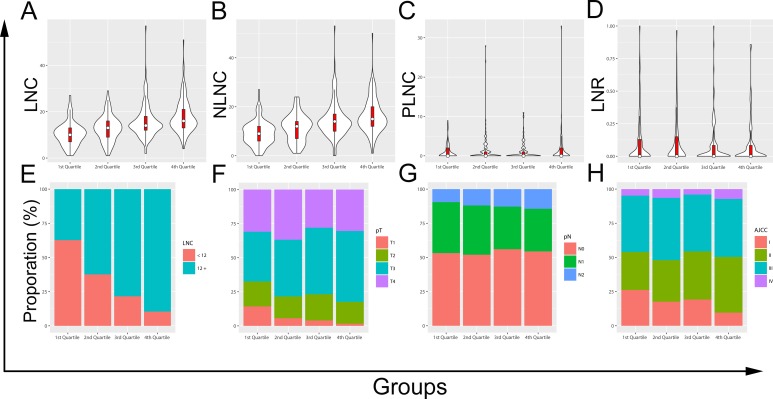
Violin plots and proportional stacked bar charts. (A) lymph node count (LNC), (B) negative lymph node count (NLNC), (C) positive lymph node count (PLNC), (D) lymph node ratio (LNR), (E) LNC, (F) pT stages, (G) pN stages, (H) American Joint Committee on Cancer (AJCC) stages.

Further comparisons using violin plots (Fig 4A–4D) revealed that increased probability of adequate lymph node recovery before surgery was associated with increased LNC and negative lymph node count (NLNC) (both *P* values for Kruskal-Wallis test < 0.001); however it did not correlated with increased positive lymph node count (PLNC) or increased lymph node ratio (LNR) (*P* values for one-Way Anova were 0.448 and 0.500). Additional assessments with proportional stacked bar charts (Fig 4E–4H) indicated that the increased probability of adequate lymph node recovery before surgery was well correlative with adequate lymph node recovery after resection of CRC (*P* < 0.001). The increased probability also showed an association with deeper infiltration (*P* = 0.004) but did not lead to apparent migrations of the pN (*P* = 0.856) or AJCC stages (*P* = 0.089).

## Discussion

In the current study, we retrospectively identified a number of predictors for adequate lymph node recovery through univariate and multivariate logistic analyses. We also developed and internally validated a preoperative nomogram, which incorporated several operator-independent markers and achieved a moderate predictive accuracy. In addition, we demonstrated with the nomogram that increased probability of adequate lymph node recovery before surgery was associated with increased LNC and NLNC during pathological assessment but it did not lead to significant changes in PLNC, LNR, pN stages or AJCC stages.

Previous researches have investigated and identified numerous clinical-pathological factors that significantly correlate with LNC. The spectrum of related variables in patients with CRC is generally wide, which includes patient-related, tumor-specific, technique-dependent and hospital-varying factors [8–14]. In the study, we reproduced similar results for some conventionally relevant and irrelevant markers such as age, tumor location, diameter, histology, type of surgery and pathologist [12, 13, 25–27]. The related mechanisms have been well explained in some tailored reviews [12, 13]. We also identified some unreported biomarkers that might be inexpensive predictors for adequate lymph node recovery before surgery.

First of all, the results of multivariate analysis agree with the consensus that the recovery of lymph node may be multifactorial. However, the significant correlation with circulating markers is inconsistent with the consideration of using LNC as an indicator for surgical or pathological quality. According to the latest NCCN guideline [3], adjuvant therapy is no longer recommended for T3N0M0 colon cancer with a MSI phenotype irrespective of the presence of any high-risk features including a LNC < 12. This update is based on clinical trials showing that stage II MSI patients have good prognosis and do not benefit from 5-FU adjuvant therapy [3]. Some pathological comparisons show that MSI CRCs are frequently present in patients with a younger age and a proximal location [28, 29], both of which were also associated with increased LNC in our study. MSI CRCs tend to represent poor differentiation due to a higher prevalence of mixed phenotypes and dominant mucinous morphology [28], a marker for increased LNC implicated by our results as well. Importantly, MSI CRCs induces more prominent lymphocytic reactions with an increased density of cytotoxic T cells which are responsible for antitumor immunity and elimination of metastasis in lymph nodes [28, 30]. The study by Ogino and colleagues also demonstrates that increased lymphocytic reaction score is not only associated with MSI CRC but also concurrently associated with increased NLNC and a tendency of increased LNC [29]. The effects of lymphocytic reactions to CRC partially explain the good prognosis of MSI CRC and may simultaneously serve as a mechanism of the association between increased LNC and prolonged survival. These findings suggest that LNC may be an indicator of lymphocytic reaction to CRC; meanwhile, the factors that correlate with a higher LNC are also involved in enhanced lymphocytic reaction. Secondly, a negative association between CEA, CA19-9, circulating neutrophils, lymphocytes and LNC was identified by our analyses. The relationship between CEA, CA19-9 and LNC may be explained as a reflection of the antagonistic balance between tumor expansion and host immunity such as lymphocytic reactions. Peripheral neutrophils and neutrophil to lymphocyte ratio (NLR) are classic markers for systemic inflammatory response (SIR) which is usually measured by modified Glasgow Prognostic Score (mGPS) [31]. Interleukin-6 gets involved in multiple processes during SIR, i.e., by inducing production of acute-phase proteins, proliferation of neutrophils and differentiation of megakaryocyte to platelets [32, 33]. Activated SIR interrelates increased c-reactive protein, circulating neutrophils, NLR, mGPS, IL-6, platelets, tumor necrosis and decreased albumin, lymphocytes, hemoglobin, peritumoral infiltrate, and of course poorer survival [32–35]. It is in accordance with a recent study which verifies that increased Foxp3^+^ tumor infiltrating lymphocytes are related to increased serum albumin and better outcomes in stage II and III CRC [36]. This also seems to be consistent with another study which finds that reduced LNC is linked to colon cancer patients with activated SIR [37]. But due to a lack of multivariate analysis, the conclusion in the study needs further validation. Our study displayed that LNC might positively correlated with platelet count. A plausible explanation is that the differentiation of megakaryocyte to platelets can be induced by tumor cells through secretion of vascular endothelial cell growth factor (VEGF) [38]. This is confirmed by another research which clarified significant associations between increased platelets and increased tumor diameter, younger age as well as higher pT stages [39]. All of the latter three variables are markers of increased LNC. With respect to lymphocyte count, we only observed a borderline association with LNC. The result may reflect either the heterogeneity of circulating lymphocytes or the less prominent importance relative to other covariates. Further studies on subpopulations of lymphocytes in the bloodstream and tumor draining lymph nodes may provide potential mechanisms between circulating, infiltrating lymphocytes and LNC [40, 41]. As for tumor deposit, its inverse relationship with LNC demonstrated in our study supports the view that metastases in the deposits and the lymph nodes are different entities, and this relationship may offer an additional reason for LNC-related survival advantage and favor the rationality of a separated N1c stage for the presence of the tumor deposit. Lastly, our nomogram was developed to preoperatively assess the probability of adequate lymph node recovery following standard surgical and pathological procedures. It is intriguing that the nomogram-predicted probability exhibited good correlation with both increased LNC and NLNC but neither PLNC nor LNR. In theory, removal of NLNC is not pathologically consequential, whilst increased NLNC has been proposed as an independent predictor for improved survival of patients with CRC [42]. Obviously, micrometastasis in pN0 patients should be a responsible mechanism because it is not a rare event. Marked regional lymphocytic reaction to CRC leads to better survival with increased LNC and NLNC as indictors, thus it may be another reason for survival advantage with NLNC. In addition, the data did not support significant stage migrations by increased LNC.

Our study has some limitations that allow us to interpret with caution. The research data were retrospectively collected and processed, during which the influence of selection bias might be underestimated. The cut-off values of circulating markers such as neutrophils and lymphocytes might be suboptimal due to a relatively small study population. Although the nomogram performed well in derivation cohort and remained stable during internal validation, it still needs independent external validation and attentions should be paid to the clinical validity of the nomogram during the external validation. Incorporation of new markers such as circulating tumor cells and cell-free DNAs may further promote the performance of the nomogram.

## Conclusions

In summary, the results of the study conclude some inexpensive and useful circulating markers that are capable of predicting the probability of adequate lymph node recovery before resection of CRC. The nomogram on the basis of the biomarkers exhibits promising performance that may allow for future validation and application in clinical practice. The identified predictors for LNC may also offer a number of new explanations of the association between increased LNC and prolonged survival. These explanations may involve the MSI phenotype, regional lymphocytic reaction, SIR, micrometastasis and host-tumor interactions under the background of cytokine imbalance. Moreover, it is likely that enhanced lymphocytic reactions are associated with improved survival by eliminating metastasis in lymph nodes and result in the increase of identifiable LNC and NLNC simultaneously. Although some studies have demonstrated that the prognostic value of LNR in CRC is superior to that of LNC, combination of LNC and LNR may achieve more prominent prognostic significance considering they are independent with each other. Next, the nomogram itself indicates that LNC is predictable. Under the context of homogeneous surgical procedures and pathological manipulations, the LNC retrieved from patients may mainly reflect patient-specific factors such as antitumor immunity and age at diagnosis rather than type of surgery or quality of pathologist. However, our study still supports a LNC ≥ 12 to be the benchmark for surgically-treated patients with CRC; because the benchmark remains evidence-based and is helpful for the standardization of surgical and pathological procedures. Eventually, the nomogram still needs external validation. Inclusion of circulating markers relating to SIR, antitumor immune response and tumor burdens may be directions of future studies.

## Supporting Information

S1 Table The STROBE checklists. (DOCX)

## References

[1] Nagtegaal ID. Current concepts of colorectal cancer resection pathology. Histopathology. 2015;66(1):102–11. 10.1111/his.12563 25263073

[2] Schmoll HJ, Van Cutsem E, Stein A, Valentini V, Glimelius B, Haustermans K, et al ESMO Consensus Guidelines for management of patients with colon and rectal cancer. a personalized approach to clinical decision making. Ann Oncol. 2012;23(10):2479–516. 10.1093/annonc/mds236 23012255

[3] Denlinger CS, Ligibel JA, Are M, Baker KS, Broderick G, Demark-Wahnefried W, et al NCCN Guidelines Insights: Survivorship, Version 1.2016. J Natl Compr Canc Netw. 2016;14(6):715–24. 27283164 10.6004/jnccn.2016.0073 PMC5865597

[4] Quirke P, Risio M, Lambert R, von Karsa L, Vieth M. Quality assurance in pathology in colorectal cancer screening and diagnosis-European recommendations. Virchows Arch. 2011;458(1):1–19. 10.1007/s00428-010-0977-6 21061133 PMC3016207

[5] Compton CC, Greene FL. The staging of colorectal cancer: 2004 and beyond. CA Cancer J Clin. 2004;54(6):295–308. 15537574 10.3322/canjclin.54.6.295

[6] Compton CC, Fielding LP, Burgart LJ, Conley B, Cooper HS, Hamilton SR, et al Prognostic factors in colorectal cancer. College of American Pathologists Consensus Statement 1999. Arch Pathol Lab Med. 2000;124(7):979–94. 10.1043/0003-9985(2000)124<0979:PFICC>2.0.CO;2 10888773

[7] Lykke J, Roikjaer O, Jess P, Danish Colorectal Cancer G. The relation between lymph node status and survival in Stage I-III colon cancer: results from a prospective nationwide cohort study. Colorectal Dis. 2013;15(5):559–65. 10.1111/codi.12059 23061638

[8] Hari DM, Leung AM, Lee JH, Sim MS, Vuong B, Chiu CG, et al AJCC Cancer Staging Manual 7th edition criteria for colon cancer: do the complex modifications improve prognostic assessment? J Am Coll Surg. 2013;217(2):181–90. 10.1016/j.jamcollsurg.2013.04.018 23768788 PMC4657944

[9] Moore J, Hyman N, Callas P, Littenberg B. Staging error does not explain the relationship between the number of lymph nodes in a colon cancer specimen and survival. Surgery. 2010;147(3):358–65. 10.1016/j.surg.2009.10.003 19962166

[10] Wong JH, Lum SS, Morgan JW. Lymph node counts as an indicator of quality at the hospital level in colorectal surgery. J Am Coll Surg. 2011;213(2):226–30. 10.1016/j.jamcollsurg.2011.05.003 21641833

[11] Sjo OH, Merok MA, Svindland A, Nesbakken A. Prognostic impact of lymph node harvest and lymph node ratio in patients with colon cancer. Dis Colon Rectum. 2012;55(3):307–15. 10.1097/DCR.0b013e3182423f62 22469798

[12] Willaert W, Mareel M, Van De Putte D, Van Nieuwenhove Y, Pattyn P, Ceelen W. Lymphatic spread, nodal count and the extent of lymphadenectomy in cancer of the colon. Cancer Treat Rev. 2014;40(3):405–13. 10.1016/j.ctrv.2013.09.013 24126120

[13] Li Destri G, Di Carlo I, Scilletta R, Scilletta B, Puleo S. Colorectal cancer and lymph nodes: the obsession with the number 12. World J Gastroenterol. 2014;20(8):1951–60. 10.3748/wjg.v20.i8.1951 24587671 PMC3934465

[14] Budde CN, Tsikitis VL, Deveney KE, Diggs BS, Lu KC, Herzig DO. Increasing the number of lymph nodes examined after colectomy does not improve colon cancer staging. J Am Coll Surg. 2014;218(5):1004–11. 10.1016/j.jamcollsurg.2014.01.039 24661856

[15] Chapman B, Paquette C, Tooke C, Schwartz M, Osler T, Weaver D, et al Impact of Schwartz enhanced visualization solution on staging colorectal cancer and clinicopathological features associated with lymph node count. Dis Colon Rectum. 2013;56(9):1028–35. 10.1097/DCR.0b013e31829c41ba 23929011

[16] Chen L, Kalady MF, Goldblum J, Seyidova-Khoshknabi D, Burks EJ, Roberts PL, et al Does reevaluation of colorectal cancers with inadequate nodal yield lead to stage migration or the identification of metastatic lymph nodes? Dis Colon Rectum. 2014;57(4):432–7. 10.1097/DCR.0000000000000052 24608298

[17] Liang JT, Lai HS, Huang J, Sun CT. Long-term oncologic results of laparoscopic D3 lymphadenectomy with complete mesocolic excision for right-sided colon cancer with clinically positive lymph nodes. Surg Endosc. 2015;29(8):2394–401. 10.1007/s00464-014-3940-9 25384361

[18] Bertelsen CA, Neuenschwander AU, Jansen JE, Wilhelmsen M, Kirkegaard-Klitbo A, Tenma JR, et al Disease-free survival after complete mesocolic excision compared with conventional colon cancer surgery: a retrospective, population-based study. Lancet Oncol. 2015;16(2):161–8. 10.1016/S1470-2045(14)71168-4 25555421

[19] Killeen S, Mannion M, Devaney A, Winter DC. Complete mesocolic resection and extended lymphadenectomy for colon cancer: a systematic review. Colorectal Dis. 2014;16(8):577–94. 10.1111/codi.12616 24655722

[20] Bui L, Rempel E, Reeson D, Simunovic M. Lymph node counts, rates of positive lymph nodes, and patient survival for colon cancer surgery in Ontario, Canada: a population-based study. J Surg Oncol. 2006;93(6):439–45. 10.1002/jso.20499 16615148

[21] Belt EJ, te Velde EA, Krijgsman O, Brosens RP, Tijssen M, van Essen HF, et al High lymph node yield is related to microsatellite instability in colon cancer. Ann Surg Oncol. 2012;19(4):1222–30. 10.1245/s10434-011-2091-7 21989661 PMC3309135

[22] Popat S, Hubner R, Houlston RS. Systematic review of microsatellite instability and colorectal cancer prognosis. J Clin Oncol. 2005;23(3):609–18. 10.1200/JCO.2005.01.086 15659508

[23] Saha S, Shaik M, Johnston G, Saha SK, Berbiglia L, Hicks M, et al Tumor size predicts long-term survival in colon cancer: an analysis of the National Cancer Data Base. Am J Surg. 2015;209(3):570–4. 10.1016/j.amjsurg.2014.12.008 25601557

[24] The Helsinki Declaration of the World Medical Association (WMA). Ethical principles of medical research involving human subjects. Pol Merkur Lekarski. 2014;36(215):298–301. 24964504

[25] Mathis KL, Green EM, Sargent DJ, Delaney C, Simmang CL, Nelson H. Surgical quality surrogates do not predict colon cancer survival in the setting of technical credentialing: a report from the prospective COST trial. Ann Surg. 2013;257(1):102–7. 10.1097/SLA.0b013e318260a8e6 23059506

[26] Jakub JW, Russell G, Tillman CL, Lariscy C. Colon cancer and low lymph node count: who is to blame? Arch Surg. 2009;144(12):1115–20. 10.1001/archsurg.2009.210 20026828

[27] Nash GM, Row D, Weiss A, Shia J, Guillem JG, Paty PB, et al A predictive model for lymph node yield in colon cancer resection specimens. Ann Surg. 2011;253(2):318–22. 10.1097/SLA.0b013e318204e637 21169808

[28] De Smedt L, Lemahieu J, Palmans S, Govaere O, Tousseyn T, Van Cutsem E, et al Microsatellite instable vs stable colon carcinomas: analysis of tumour heterogeneity, inflammation and angiogenesis. Br J Cancer. 2015;113(3):500–9. 10.1038/bjc.2015.213 26068398 PMC4522625

[29] Ogino S, Nosho K, Irahara N, Meyerhardt JA, Baba Y, Shima K, et al Lymphocytic reaction to colorectal cancer is associated with longer survival, independent of lymph node count, microsatellite instability, and CpG island methylator phenotype. Clin Cancer Res. 2009;15(20):6412–20. 10.1158/1078-0432.CCR-09-1438 19825961 PMC2771425

[30] Drescher KM, Sharma P, Watson P, Gatalica Z, Thibodeau SN, Lynch HT. Lymphocyte recruitment into the tumor site is altered in patients with MSI-H colon cancer. Fam Cancer. 2009;8(3):231–9. 10.1007/s10689-009-9233-0 19165625

[31] Roxburgh CS, Wallace AM, Guthrie GK, Horgan PG, McMillan DC. Comparison of the prognostic value of tumour- and patient-related factors in patients undergoing potentially curative surgery for colon cancer. Colorectal Dis. 2010;12(10):987–94. 10.1111/j.1463-1318.2009.01961.x 19555389

[32] Ishizuka M, Nagata H, Takagi K, Iwasaki Y, Kubota K. Combination of platelet count and neutrophil to lymphocyte ratio is a useful predictor of postoperative survival in patients with colorectal cancer. Br J Cancer. 2013;109(2):401–7. 10.1038/bjc.2013.350 23820256 PMC3721384

[33] Guthrie GJ, Roxburgh CS, Horgan PG, McMillan DC. Does interleukin-6 link explain the link between tumour necrosis, local and systemic inflammatory responses and outcome in patients with colorectal cancer? Cancer Treat Rev. 2013;39(1):89–96. 10.1016/j.ctrv.2012.07.003 22858249

[34] Roxburgh CS, Salmond JM, Horgan PG, Oien KA, McMillan DC. Comparison of the prognostic value of inflammation-based pathologic and biochemical criteria in patients undergoing potentially curative resection for colorectal cancer. Ann Surg. 2009;249(5):788–93. 10.1097/SLA.0b013e3181a3e738 19387324

[35] Guthrie GJ, Roxburgh CS, Richards CH, Horgan PG, McMillan DC. Circulating IL-6 concentrations link tumour necrosis and systemic and local inflammatory responses in patients undergoing resection for colorectal cancer. Br J Cancer. 2013;109(1):131–7. 10.1038/bjc.2013.291 23756867 PMC3708575

[36] Wang DL, Liu YY, Gu YL, Qin Y, Ji HF, Wu LH, et al Increased number of forkhead box P3+ tumor-infiltrating lymphocytes correlates with high preoperative albumin level and better survival in patients with stage II or III colorectal cancer. Tumour Biol. 2015;36(7):5407–14. 10.1007/s13277-015-3206-8 25697896

[37] Murphy B, Kennelly R, Yousef H, Mehigan B, McCormick P. Activated systemic inflammatory response at diagnosis reduces lymph node count in colonic carcinoma. Int J Surg. 2014;183:S23–S. 10.4251/wjgo.v8.i8.623 PMC4980653 27574555

[38] Troxler M, Dickinson K, Homer-Vanniasinkam S. Platelet function and antiplatelet therapy. Br J Surg. 2007;94(6):674–82. 10.1002/bjs.5852 17514662

[39] Sasaki K, Kawai K, Tsuno NH, Sunami E, Kitayama J. Impact of preoperative thrombocytosis on the survival of patients with primary colorectal cancer. World J Surg. 2012;36(1):192–200. 10.1007/s00268-011-1329-7 22045447

[40] Ling KL, Pratap SE, Bates GJ, Singh B, Mortensen NJ, George BD, et al Increased frequency of regulatory T cells in peripheral blood and tumour infiltrating lymphocytes in colorectal cancer patients. Cancer Immun. 2007;7:7 17388261 PMC2935744

[41] Xu B, Yuan L, Gao Q, Yuan P, Zhao P, Yuan H, et al Circulating and tumor-infiltrating Tim-3 in patients with colorectal cancer. Oncotarget. 2015;6(24):20592–603. 10.18632/oncotarget.4112 26008981 PMC4653028

[42] Johnson PM, Porter GA, Ricciardi R, Baxter NN. Increasing negative lymph node count is independently associated with improved long-term survival in stage IIIB and IIIC colon cancer. J Clin Oncol. 2006;24(22):3570–5. 10.1200/JCO.2006.06.8866 16877723

