# Supplementary material for: A Nomogram to Predict Adequate Lymph Node Recovery before Resection of Colorectal Cancer
Source: PLoS One. 2016 Dec 16;11(12):e0168156. doi: 10.1371/journal.pone.0168156 (PMC5161509; doi:10.1371/journal.pone.0168156)
Supplement: S1 Table — (DOCX) [file pone.0168156.s001.docx]

STROBE Statement—checklist of items that should be included in reports of observational studies

|  | Item No. | Recommendation | Page  No. | Relevant text from manuscript |
| --- | --- | --- | --- | --- |
| **Title and abstract** | 1 | (*a*) Indicate the study’s design with a commonly used term in the title or the abstract | 2 | The study design was indicated as retrospective in the method descriptions of the abstract. |
|  |  | (*b*) Provide in the abstract an informative and balanced summary of what was done and what was found | 2 | All major analyses and findings were described in the abstract. Refer to the abstract for details. |
| Introduction | | | |  |
| Background/rationale | 2 | Explain the scientific background and rationale for the investigation being reported | 3 to 5 | Please refer to the first and second paragraphs of the **Introduction** section. |
| Objectives | 3 | State specific objectives, including any prespecified hypotheses | 5 | The primary and secondary aims of the study were specified in the third paragraph of the **Introduction** section. |
| Methods | | | |  |
| Study design | 4 | Present key elements of study design early in the paper | 5 | The first sentence of the Materials and Methods section indicated the study design as retrospective. |
| Setting | 5 | Describe the setting, locations, and relevant dates, including periods of recruitment, exposure, follow-up, and data collection | 5 and 6 | Details were described in the first two paragraphs of the Materials and Methods section. |
| Participants | 6 | (*a*) *Cohort study*—Give the eligibility criteria, and the sources and methods of selection of participants. Describe methods of follow-up  *Case-control study*—Give the eligibility criteria, and the sources and methods of case ascertainment and control selection. Give the rationale for the choice of cases and controls  *Cross-sectional study*—Give the eligibility criteria, and the sources and methods of selection of participants | 5 and 6 | Details were described in the first paragraph of the Materials and Methods section. Inclusion and exclusion criteria were given along with the number of excluded patients at each step. The study did not involve follow-up information. |
|  |  | (*b*) *Cohort study*—For matched studies, give matching criteria and number of exposed and unexposed  *Case-control study*—For matched studies, give matching criteria and the number of controls per case | Not applicable. | Not applicable. |
| Variables | 7 | Clearly define all outcomes, exposures, predictors, potential confounders, and effect modifiers. Give diagnostic criteria, if applicable | 6 and 7 | Please refer to the second paragraph of the Patients and variables section and the first paragraph of the Statistical methods section for variables and outcome information. |
| Data sources/ measurement | 8* | For each variable of interest, give sources of data and details of methods of assessment (measurement). Describe comparability of assessment methods if there is more than one group | 5 to 8 | The source of data was indicated. Others were not applicable. |
| Bias | 9 | Describe any efforts to address potential sources of bias | 7 to 8 | The study used multivariate analysis, bootstrapping calibration and validation to yield adjusted effects and bias-corrected estimates. |
| Study size | 10 | Explain how the study size was arrived at | 5 to 6 | Consecutive patients were considered. Ineligible patients were excluded before analysis according to detailed exclusion criteria. |

Continued on next page

| Quantitative variables | 11 | Explain how quantitative variables were handled in the analyses. If applicable, describe which groupings were chosen and why | 7 | Concentrations of blood markers were pre-categorized to predict LNC ≥ 12 according to their normal references (i.e., for Hb and PLT) or optimal cut-off values determined by maximization of Yuden Index with receiver operating characteristic (ROC) curve analysis, based on clinical reasoning and significance. Quantitative tumor factors were not categorized to avoid information loss. |
| --- | --- | --- | --- | --- |
| Statistical methods | 12 | (*a*) Describe all statistical methods, including those used to control for confounding | 7 and 8 | Please refer to the Statistical methods section for details. |
|  |  | (*b*) Describe any methods used to examine subgroups and interactions | 7 and 8 | Subgroups and interactions analyses were not applicable for the study. |
|  |  | (*c*) Explain how missing data were addressed | 7 and 8 | Patients with miss data were excluded before analysis. |
|  |  | (*d*) *Cohort study*—If applicable, explain how loss to follow-up was addressed  *Case-control study*—If applicable, explain how matching of cases and controls was addressed  *Cross-sectional study*—If applicable, describe analytical methods taking account of sampling strategy | Not applicable. | Not applicable. |
|  |  | (*e*) Describe any sensitivity analyses | Not applicable. | Not applicable. |
| Results | | | | |
| Participants | 13* | (a) Report numbers of individuals at each stage of study—eg numbers potentially eligible, examined for eligibility, confirmed eligible, included in the study, completing follow-up, and analysed | 5 and 6 | The inclusion and exclusion of patients with detailed numbers were described in the first paragraph of the Patients and variables section. |
|  |  | (b) Give reasons for non-participation at each stage | 5 and 6 | Please see above. |
|  |  | (c) Consider use of a flow diagram | 5 and 6 | Flow diagram of study development was not used because there was only one study group. The inclusion and exclusion criteria were described in detailed instead of a flow diagram. |
| Descriptive data | 14* | (a) Give characteristics of study participants (eg demographic, clinical, social) and information on exposures and potential confounders | 8 to 10 | Please refer to Table 1. |
|  |  | (b) Indicate number of participants with missing data for each variable of interest | Not applicable. | No miss data in the study. |
|  |  | (c) *Cohort study*—Summarise follow-up time (eg, average and total amount) | Not applicable. | No follow-ups in the study. |
| Outcome data | 15* | *Cohort study*—Report numbers of outcome events or summary measures over time | Not applicable. | Not applicable. |
|  |  | *Case-control study—*Report numbers in each exposure category, or summary measures of exposure | Not applicable. | Not applicable. |
|  |  | *Cross-sectional study—*Report numbers of outcome events or summary measures | Not applicable. | Not applicable. |
| Main results | 16 | (*a*) Give unadjusted estimates and, if applicable, confounder-adjusted estimates and their precision (eg, 95% confidence interval). Make clear which confounders were adjusted for and why they were included | 11 and 12 | Results of unadjusted and adjusted estimates were shown in Table 2 and 3, respectively. Specific reasons were provided. |
|  |  | (*b*) Report category boundaries when continuous variables were categorized | 11 and 12 | Please refer to Tables 1 to 3 for details. |
|  |  | (*c*) If relevant, consider translating estimates of relative risk into absolute risk for a meaningful time period | Not applicable. | Not applicable. |

Continued on next page

| Other analyses | 17 | Report other analyses done—eg analyses of subgroups and interactions, and sensitivity analyses | Not applicable. | Not applicable. |
| --- | --- | --- | --- | --- |
| Discussion | | | | |
| Key results | 18 | Summarise key results with reference to study objectives | 15 to 18 | Please refer to the first three paragraphs of the Discussion section. |
| Limitations | 19 | Discuss limitations of the study, taking into account sources of potential bias or imprecision. Discuss both direction and magnitude of any potential bias | 18 and 19 | Please refer to the last paragraph of the Discussion section. Relevant limitations including selection bias were discussed. |
| Interpretation | 20 | Give a cautious overall interpretation of results considering objectives, limitations, multiplicity of analyses, results from similar studies, and other relevant evidence | 15 to 18, 19 and 20 | Please refer to the Discussion section and Conclusion section. |
| Generalisability | 21 | Discuss the generalisability (external validity) of the study results | 15 to 18, 19 and 20 | Please refer to the first three paragraphs of the Discussion section, and the conclusion section for details. The nomogram on the basis of the biomarkers exhibits promising performance that may allow for future validation and application in clinical practice. |
| Other information | |  | | |
| Funding | 22 | Give the source of funding and the role of the funders for the present study and, if applicable, for the original study on which the present article is based | Page 2 of the PDF version for review. | The funding sources including the role in the current study were indicated. |

*Give information separately for cases and controls in case-control studies and, if applicable, for exposed and unexposed groups in cohort and cross-sectional studies.

**Note:** An Explanation and Elaboration article discusses each checklist item and gives methodological background and published examples of transparent reporting. The STROBE checklist is best used in conjunction with this article (freely available on the Web sites of PLoS Medicine at http://www.plosmedicine.org/, Annals of Internal Medicine at http://www.annals.org/, and Epidemiology at http://www.epidem.com/). Information on the STROBE Initiative is available at www.strobe-statement.org.
